# Paternal ischemic heart disease and chance of successful pregnancy outcomes

**DOI:** 10.1111/andr.70065

**Published:** 2025-05-22

**Authors:** Anne‐Sofie Sønnichsen‐Dreehsen, Caroline Theilgaard Thorarinsson, Bente Mertz Nørgård, Jens Fedder, Mette Wod

**Affiliations:** ^1^ Centre of Andrology & Fertility Clinic Odense University Hospital & University of Southern Denmark Odense Denmark; ^2^ Center for Clinical Epidemiology Odense University Hospital Odense Denmark; ^3^ Research Unit of Clinical Epidemiology Department of Clinical Research University of Southern Denmark Odense Denmark

**Keywords:** clinical epidemiology, coronary artery disease, coronary heart disease, ischemic heart disease, paternal, pregnancy

## Abstract

**Background:**

Only approximately 30% of conceptions result in live births. Historically, research has predominantly focused on maternal factors impacting pregnancy success, despite the cause remaining unidentified in most cases. The influence of paternal factors on a couple's likelihood of achieving a successful pregnancy is still not well understood and warrants further investigation.

**Objectives:**

This study aims to examine the chance of biochemical pregnancy, clinical pregnancy, and a live‐born child in couples where the male partner has ischemic heart disease.

**Materials and methods:**

This nationwide cohort study based on Danish health registries included couples undergoing in vitro fertilization with or without intracytoplasmic sperm injection from 2006 to 2019. The cohort was divided into two groups: exposed and unexposed. The exposed cohort included embryo transfers in couples where the male partner had ischemic heart disease, while the unexposed group included those where the male partner did not have this condition.

**Results:**

A total of 101,875 couples with a known male partner were included. Among these, 653 couples were included in the exposed cohort and 101,222 were included in the unexposed cohort. The adjusted odd ratios (ORs) for a biochemical pregnancy, clinical pregnancy, and live‐born child were 0.99 (95% confidence interval [CI]: 0.79; 1.23), 0.79 (95% CI: 0.51, 1.23), and 0.94 (95% CI: 0.62, 1.44), respectively.

**Conclusions:**

These findings indicate that paternal ischemic heart disease prior to oocyte retrieval is not associated with a statistically significant decrease in the chances of biochemical pregnancy, clinical pregnancy, or live birth.

## 1 INTRODUCTION

Assisted reproductive technology (ART) treatments are increasingly utilized, with an estimated 10–20% of couples experiencing unwanted infertility.^1^ In Denmark, ART procedures contribute to approximately 12% of annual births.^2^ Unwanted infertility, characterized by failure to conceive after 12 months of trying, or recurrent miscarriages imposes significant psychological distress on affected couples.^1^, ^3^ The cause of infertility can be attributed to female factors in approximately one‐third of cases, paternal factors in about one‐third of cases, and both female and male factors or unexplained in about one‐third of cases.^4^ Research indicates that chromosomal abnormalities in the fetus are the most common cause of pregnancy loss during the first 10 weeks of gestation.^5^ Maternal factors such as advanced maternal age, anatomical abnormalities, autoimmune disorders, and chromosomal changes have been associated with an increased risk of pregnancy loss.^6^ Additionally, lifestyle factors in females, including alcohol intake, smoking, obesity, and caffeine consumption, have also been linked to a higher risk of unwanted pregnancy and birth outcomes.^7^, ^8^, ^9^ Despite extensive research on various maternal etiologies, the cause of pregnancy loss remains unidentified in most cases.^10^, ^11^ This has led to a growing focus on the potential impact of paternal factors on the success of pregnancy.^12^, ^13^, ^14^ However, the evidence in this area is still limited.^14^ Paternal lifestyle factors, such as smoking and education level, have been found to influence male fertility and pregnancy outcomes.^15^, ^16^ Varicocele, metabolic syndrome, and cardiovascular diseases in males have all been suggested to be associated with adverse pregnancy outcomes.^17^, ^18^, ^19^, ^20^ A large study on 958,804 pregnancies using US claims data has shown an increased risk of pregnancy loss among couples where the male partner had multiple comorbidities,^18^ indicating that male health may significantly impact a couple's chance of achieving a successful pregnancy. Ischemic heart disease is one of the leading causes of death globally^21^ and is characterized by atherosclerosis or atherosclerotic occlusions of the coronary arteries and is recognized as a chronic inflammatory disease.^22^, ^23^ Further exploration of the relationship between ischemic heart disease and other male health conditions and fertility outcomes is warranted, given their potential consequences for reproductive health. In this nationwide cohort study, we aimed to examine whether couples, where the male partner had ischemic heart disease, have a reduced chance of successful pregnancy outcomes compared with couples where the male partner did not have ischemic heart disease. This was done by estimating the chance of (1) biochemical pregnancy, (2) clinical pregnancy, and (3) a live‐born child.

## 2 MATERIALS AND METHODS

We conducted a nationwide cohort study using Danish national health registries. The study focused on couples undergoing ART treatment from 2006 to 2019 (in vitro fertilization [IVF] or intracytoplasmic sperm injection [ICSI] treatments) and involved both fresh cycle embryo replacement transfers and frozen embryo replacement (FER) transfers.

The study population was followed closely during their treatment. Pregnancy tests were performed two weeks after every embryo transfer, followed by a pregnancy scan three weeks after a positive pregnancy test. This allowed complete follow‐up on all pregnancy events.

### 2.1 The study population and exposure

The study cohort comprised all couples in the Danish ART registry^24^, ^25^, ^26^ who underwent embryo transfers between January 1, 2006 and September 3, 2019. In Denmark, infertile couples and single women are eligible for up to three fresh fertility treatment cycles if the woman is under 41 years old at treatment initiation. Although the Danish ART registry has existed since 1994, we focused solely on data from 2006 onwards due to insufficient information regarding embryo transfers utilizing the FER method between 1994 and 2005. We divided couples into two groups: exposed and unexposed. Couples were considered exposed if the male partner had been diagnosed with ischemic heart disease before the oocyte retrieval procedure. We identified male partners with ischemic heart disease using specific medical codes (ICD‐10 codes I20‐I25) and required at least one hospital diagnosis before the oocyte retrieval to include them in the exposed group. Couples who did not meet these criteria were placed in the unexposed group.

We excluded couples who used donor sperm or oocytes, as well as couples who did not undergo IVF or ICSI treatments, such as hormone therapy, testicular sperm retrieval, percutaneous epididymal sperm aspiration, and uterine insemination. We also excluded couples where the treatments resulted in multiple pregnancies If a couple went through more than one oocyte retrieval procedure, and the male partner was diagnosed with ischemic heart disease between two of these procedures, they could be considered for inclusion in both the exposed and unexposed groups.

### 2.2 Data sources

Several Danish health registries were used for this study. Information on the Danish population, including migration, civil registration number, and death, was available in the Danish Civil Registration System.^27^ The civil registration number can be used to link information from the different population registers included in this study since it is used across all Danish health registries. Information on all ART procedures performed in public and private clinics is recorded in the Danish ART registry, including embryo transfers, pregnancies, and causes of infertility.^24^, ^25^, ^26^

The Medical Birth Registry provides detailed information on all births in Denmark since 1973, including data on the father, the mother, the birth, the child, and whether an ART treatment results in a live‐born child.^28^, ^29^ The National Patient Registry contains data on underlying diseases of all Danish patients who have been hospitalized, including diagnoses based on the International Classification of Diseases (ICD), with ICD‐10 being used since 1994.^30^ Data on medication was obtained from the Danish National Prescription Registry which contains data on all redeemed prescriptions in Denmark since 1995.^31^

### 2.3 Statistical analysis

Multilevel logistic regression analysis, with a 95% confidence interval (CI), was used to estimate both crude and adjusted odds ratios (OR) for the likelihood of biochemical pregnancy, clinical pregnancy, and live birth among couples with male partners diagnosed with ischemic heart disease compared to those without such a diagnosis. Multilevel regression was used to account for multiple embryo transfers for each couple during the study period.

Possible covariates were defined and tested a priori. The final model included the following covariates: female age (continuous): Age of the female partner at the time of treatment initiation, male age (continuous): age of the male partner at the time of treatment initiation, female body mass index (BMI): Categorized as underweight (<18.5), normal (18.5–24.99), overweight (25.00–29.99), and obese (>30), female smoking status (yes/no): defined as any smoking at the time of treatment initiation, female alcohol intake (yes/no): defined as consuming > 1 drink per week at the time of treatment initiation, female Charlson comorbidity index (CCI): presence or absence of comorbidities, classified as CCI of 0 or ≥1, male CCI: similar to female CCI, with comorbidities categorized as CCI of 0 or ≥1, calendar year of ART treatment: categorized into three periods: 2006–2009, 2010–2014, and 2015–2019, type of ART treatment: treatment types included IVF, ICSI, IVF combined with FER, and ICSI combined with FER, mother's previous history of abortions (yes/no): occurrence of previous abortions in the female partner, cause of infertility (male, female, both): categorized as solely male, solely female, or both partners contributing to infertility. Cause of infertility is coded in the Danish ART registry and is based on clinical evaluation in Danish fertility clinics. Male infertility include factors such as oligozoospermia, asthenozoospermia, azoospermia, retrograde ejaculation and erectile dysfunction, female infertility includes factors such as polycystic ovarian syndrome, ovulatory dysfunction, tubal factor infertility, uterine myomas, adenomyosis, and endometriosis. Parity (0/+1) was omitted from the final model as its inclusion did not alter the findings. Additionally, due to a substantial amount of missing data, the impact of male alcohol intake (yes/no) and male smoking status (yes/no) was not included in the final model.

To examine whether medication for ischemic heart disease impacted our outcomes, we conducted a subanalysis including only couples in which the male partner had ischemic heart disease. This analysis was then categorized based on whether they received medical treatment classified under ATC group C (cardiovascular system) within the six months preceding oocyte retrieval.

All statistical analyses were performed using Stata version 18.0 (StataCorp).

### 2.4 Ethics and approvals

The study was approved by the research notification in the Region of Southern Denmark/the Danish Data Protection Agency (journal number 22/13116).

### 2.5 Patient and public involvement

Patient representatives were engaged during the initial stages of this project. All representatives were members of the research council for the department where this study was conducted.

## 3 RESULTS

We included a total of 101,875 couples undergoing IVF or ICSI from 2006 to 2019. Out of these, 653 couples had a male partner diagnosed with ischemic heart disease. Table 1 shows the basic characteristics of couples with male ischemic heart disease and couples without male ischemic heart disease. The groups were similar in terms of factors like female health, treatment year, type of treatment, pregnancy outcomes, and female BMI. In the exposed group, male‐related infertility accounted for 27.9% of couples, compared to 28.8% in the unexposed group. Female‐related infertility was 32.9% in the exposed group and 33.3% in the unexposed group. Both groups had a similar percentage of couples with a mix of factors or unknown causes of infertility. Couples who had a parity of 0 were very similar in the exposed and unexposed cohorts with 88.4% in the exposed cohort having a parity of 0 and 86.8% in the unexposed cohort. The history of pregnancy loss was similar between the exposed and unexposed cohorts as 30.6% and 31.5% had experienced earlier pregnancy loss, respectively. Males in the exposed cohort were older compared to the unexposed cohort with a median age of 41 years (36–47 interquartile range [IQR]) compared to 35 years (32–40, IQR). The crude and adjusted ORs for biochemical pregnancy, clinical pregnancy, and live births for the exposed and unexposed cohorts are given in Table 2. There was no statistically significant difference between the exposed and unexposed groups in terms of achieving biochemical pregnancy (OR 0.99; 95% CI: 0.79:1.23), clinical pregnancy (0.79; 95% CI: 0.51:1.23), and live‐born child (0.94; 95% CI: 0.62:1.44). In the subanalysis of couples in which the male partner had ischemic heart disease, a total of 653 embryo transfers were performed, of which 272 were in couples where the male partner had received treatment for cardiovascular conditions. This analysis showed no statistically significant difference between the treated and non‐treated groups (Table 3).

**TABLE 1:** Descriptive characteristics of all males and females included in the two cohorts: ischemic heart disease and unexposed in the study period of January 2, 2006 through September 3, 2019.

	Exposed cohort (embryo transfers in expecting fathers with ischemic heart disease), *n* = 653	Unexposed cohort (embryo transfers in non‐ischemic heart disease expecting fathers) *n* = 101,222
**Paternal information**		
Age at embryo aspiration, in years		
Median (IQR^a^)	41.0 (36.0–47.0)	35.0 (32.0–40.0)
Paternal CCI^b^ (10 years back from embryo aspiration)		
No comorbidity, *n* (%)	467 (71.5)	94,519 (93.4)
Some comorbidity, *n* (%)	186 (28.5)	6703 (6.6)
Duration of ischemic heart disease at the time of embryo aspiration		
Median (IQR)	4.3 (2.1–7.4)	. (.–.)
Smoking^c^		
Non‐smoker, *n* (%)	380 (58.2)	69,860 (69.0)
Smoker, *n* (%)	106 (16.2)	13,092 (12.9)
Missing, *n* (%)	167 (25.6)	18,270 (18.0)
Alcohol^c^		
No, *n* (%)	218 (33.4)	27,528 (27.2)
Yes, *n* (%)	241 (36.9)	53,357 (52.7)
Missing, *n* (%)	194 (29.7)	20,337 (20.1)
**Maternal information**		
Age at embryo aspiration, in years		
Median (IQR^a^)	36.0 (32.0–39.0)	34.0 (30.0–38.0)
Maternal CCI^b^ (10 years back from embryo aspiration)		
No comorbidity, *n* (%)	596 (91.3)	94,015 (92.9)
Some comorbidity, *n* (%)	57 (8.7)	7207 (7.1)
Parity		
0, *n* (%)	577 (88.4)	87,820 (86.8)
1+, *n* (%)	76 (11.6)	13,402 (13.2)
History of pregnancy loss (prior to embryo aspiration)		
No, *n* (%)	453 (69.4)	69,372 (68.5)
Yes, *n* (%)	200 (30.6)	31,850 (31.5)
BMI^d^		
<18.5 (underweight), *n* (%)	21 (3.2)	3126 (3.1)
18.5–24.99 (normal), *n* (%)	290 (44.4)	54,895 (54.2)
25.00–29.99 (overweight), *n* (%)	158 (24.2)	23,627 (23.3)
≥30.00 (obese), *n* (%)	75 (11.5)	9917 (9.8)
Missing, *n* (%)	109 (16.7)	9657 (9.5)
Smoking^c^		
Non‐smoker, *n* (%)	508 (77.8)	83,767 (82.8)
Smoker, *n* (%)	44 (6.7)	7640 (7.5)
Missing, *n* (%)	101 (15.5)	9815 (9.7)
Alcohol^c^		
No, *n* (%)	353 (54.1)	49,910 (49.3)
Yes, *n* (%)	170 (26.0)	39,404 (38.9)
Missing, *n* (%)	130 (19.9)	11,908 (11.8)
**Information on ART treatment** ^**e**^		
Calendar year of infertility treatment		
2006–2009, *n* (%)	189 (28.9)	28,180 (27.8)
2010–2014, *n* (%)	250 (38.3)	39,754 (39.3)
2015–2019, *n* (%)	214 (32.8)	33,288 (32.9)
Type of ART treatment		
IVF^f^	294 (45.0)	43,431 (42.9)
ICSI^g^	283 (43.3)	44,504 (44.0)
IVF+FER^h^	43 (6.6)	7377 (7.3)
ICSI+FER	33 (5.1)	5910 (5.8)
Cause of infertility, female/male factor^i^		
Female factor, *n* (%)	215 (32.9)	33,703 (33.3)
Male factor, *n* (%)	182 (27.9)	29,195 (28.8)
A mixture of factors/idiopathic, *n* (%)	220 (33.7)	34,493 (34.1)
Missing, *n* (%)	36 (5.5)	3831 (3.8)
**Information on pregnancy outcomes**		
Biochemical		
Yes, *n* (%)	222 (34.0)	38,814 (38.3)
Clinical^j^		
Yes, *n* (%)	184 (82.9)	33,201 (85.5)
Live‐born child^k^		
Yes, *n* (%)	142 (77.2)	26,462 (79.7)

^a^ Interquartile range.

^b^ Charlson comorbidity index.

^c^ At time of treatment initiation.

^d^ Body mass index.

^e^ Assisted reproductive technology.

^f^ In vitro fertilization.

^g^ Intracytoplasmic sperm injection.

^h^ Frozen embryo transfer.

^i^ Categorized as solely male, solely female, or both partners contributing to infertility.

^j^ Among those who had a positive biochemical pregnancy.

^k^ Among those who had a positive clinical pregnancy test.

**TABLE 2:** Logistic regression analysis of the chance of biochemical pregnancy, clinical pregnancy, and live birth after assisted reproductive technology (ART) treatments in women whose male partner has ischemic heart disease.

	Exposed cohort (embryo transfers in women whose partner has ischemic heart disease)	Unexposed cohort (embryo transfers in women whose partner does not have ischemic heart disease)	Crude OR (95% CI)	Adjusted OR (95% CI)^a^
Biochemical pregnancy (hCG)				
Yes, *n* (%)	222 (34.0)	38,814 (38.3)	0.80 (0.66–0.97)	0.99 (0.79–1.23)
No, *n* (%)	431 (66.0)	62,408 (61.7)		
Clinical pregnancy (ultrasound)				
Yes, *n* (%)	184 (82.9)	33,201 (85.5)	0.80 (0.54–1.19)	0.79 (0.51–1.23)
No, *n* (%)	38 (17.1)	5613 (14.5)		
Live birth				
Yes, *n* (%)	142 (77.2)	26,462 (79.7)	0.86 (0.58–1.26)	0.94 (0.62–1.44)
No, *n* (%)	42 (22.8)	6739 (20.3)		

^a^ Adjusted for mother's and father's ages at aspiration, mother's BMI, mother's alcohol intake, mother's smoking habits, mother's Charlson index, father's Charlson index, calendar year, type of ART treatment, mother's previous history of abortions, and female/male factor.

**TABLE 3:** Logistic regression analysis of the chance of biochemical pregnancy, clinical pregnancy, and live birth after assisted reproductive technology (ART) treatments in women whose male partner has ischemic heart disease.

	Exposed cohort (embryo transfers in women whose partner has ischemic heart disease and used medicine)	Unexposed cohort (embryo transfers in women whose partner has ischemic heart disease and did not use medicine)	Crude OR (95% CI)	Adjusted OR (95% CI)^a^
Biochemical pregnancy (hCG)				
Yes, *n* (%)	87 (32.0)	135 (35.4)	0.81 (0.52–1.26)	1.09 (0.64–1.84)
No, *n* (%)	185 (68.0)	246 (64.6)		
Clinical pregnancy (ultrasound)				
Yes, *n* (%)	70 (80.5)	114 (84.4)	0.67 (0.23–2.00)	1.13 (0.40–3.18)
No, *n* (%)	17 (19.5)	21 (15.6)		
Live birth				
Yes, *n* (%)	52 (74.3)	90 (78.9)	0.75 (0.34–1.67)	‐^b^
No, *n* (%)	18 (25.7)	24 (21.1)		

^a^ Adjusted for mother's and father's ages at aspiration, mother's BMI, mother's alcohol intake, mother's smoking habits, mother's Charlson index, father's Charlson index, calendar year, type of ART treatment, mother's previous history of abortions, and female/male‐factor.

^b^ Too few observations for adjustments.

## 4 DISCUSSION

This nationwide study found no association between paternal ischemic heart disease and a reduced chance of achieving a successful pregnancy. This was consistent for key reproductive outcomes, including biochemical pregnancy, clinical pregnancy, and live birth. These findings suggest that the presence of paternal ischemic heart disease does not negatively impact the reproductive success of couples undergoing ART treatment.

Mikkelsen et al. demonstrated an increased risk of myocardial infarction, stroke, and all‐cause mortality in females, but not in their male partners, among couples who had experienced prior pregnancy loss.^32^ While our study examined ischemic heart disease as an exposure rather than an outcome, we similarly found no association between unsuccessful pregnancy and ischemic heart disease. Eisenberg et al.^33^ reported an increased risk of cardiovascular disease‐related mortality among childless men, suggesting a potential link between cardiovascular health and fertility status. However, it is imperative to note that the absence of offspring in these cases does not inherently imply infertility.^34^ Thus, the lack of children may not be explained by infertility or pregnancy loss alone, as many other factors can affect reproductive outcomes. The possible association between men with cardiovascular disease and male infertility was not investigated further in the study by Eisenberg et al.^33^ In a separate study by Eisenberg et al. on 5037 fresh ART cycles, it was observed that couples wherein the male partner had circulatory disease experienced a notably higher rate of pregnancy loss (13%) compared with couples without the male circulatory disease.^19^ Previous research has explored various factors in men that may impact the risk of pregnancy loss. This suggests that the choice of treatment for certain conditions may influence a couple's likelihood of achieving a live birth. However, our study did not investigate whether different treatments for ischemic heart disease might affect the probability of successful pregnancy. Additionally, research by Banihani et al.^35^ suggested that aspirin intake could potentially decrease male fertility by impairing sperm motility and vitality. Conversely, another study by Banihani et al.^36^ found no significant impact of statins on human semen quality characteristics. Notably, aspirin and statins are commonly prescribed for ischemic heart disease treatment.^37^, ^38^

Our study has several strengths. The main strength of this study is that it is a nationwide study based on an unselected cohort of all couples undergoing ART treatment from 2006 to 2019 using Danish health registries with high validity.^24^, ^30^, ^31^ The Danish health registries are renowned for their thoroughness and high data quality, preventing the risk of loss to follow‐up.^39^ It is a strength that we used the Danish ART registry in this study as it is based on valid and complete data.^24^, ^25^, ^26^ The utilization of data from the Danish ART registry facilitated comprehensive monitoring of reproductive events, enabling a detailed examination of the influence of paternal ischemic heart disease on early pregnancy outcomes and live births. Use of these registries allowed us to include a large study population in the exploration of successful pregnancy outcomes. Additionally, the risk of information bias was minimized as data collection occurred continuously through registers, without preconceived hypotheses or outcome measurements in mind.^40^ By adjusting for a wide range of potential confounders, such as female and male CCI, type of ART treatment, and the cause of infertility, we enhance the internal validity of the study. Another strength of this study is the inclusion of a subanalysis considering medical treatment for cardiovascular conditions. Furthermore, this is the first study to examine biochemical and clinical pregnancies, as well as live births, in couples where the male partner has ischemic heart disease.

Our study also had limitations. Due to a substantial amount of missing data, adjustments for smoking status and alcohol intake in males were not feasible. Nevertheless, it is worth noting that the existing evidence regarding the association between alcohol and/or smoking habits in men and pregnancy loss is currently limited.^41^ However, this study utilizes a non‐randomized design, which, despite incorporating several covariates, has the potential for confounding due to unknown factors. While multiple confounders are accounted for in the models, the influence of unidentified confounders cannot be excluded in an observational study. Due to the limitations of registry‐based data, we lacked access to detailed information on semen parameters, hormonal profiles and metabolic profile. The absence of metabolic data, in particular, is a limitation of this study. Further research incorporating these factors could provide valuable insights. Furthermore, our study population comprised couples undergoing ART treatment, and our results may not be directly comparable to couples attempting conception without ART treatment. However, these couples share similar conditions for achieving pregnancy, apart from differences related to ischemic heart disease.

## 5 CONCLUSIONS

The findings of this study indicate that paternal ischemic heart disease does not reduce the chance of a couple achieving a biochemical pregnancy, clinical pregnancy, or the birth of a live child compared to couples unaffected by paternal ischemic heart disease. However, further studies are needed to assess the impact of lifestyle factors, such as smoking habits and alcohol intake, before definitive conclusions can be made. Additionally, research should be conducted in non‐ART populations and focus on the effects of medical treatments to provide a more comprehensive understanding of how these variables influence fertility outcomes.

It is important to continue investigating how paternal health issues may influence couples’ chances of successful pregnancies as it could help clinicians in their consultations with patients struggling with fertility issues.

## AUTHOR CONTRIBUTIONS

All authors contributed to the conception, design, data collection, analysis, and interpretation. They all participated in drafting the article or revising it critically for significant intellectual content, approved the final version for publication, and agreed to be accountable for all aspects of the work.

## CONFLICT OF INTEREST STATEMENT

The authors declare no conflicts of interest.

## Data Availability

Data sharing is not applicable to this article as no new data were created or analyzed in this study.
